# The Impact of Fermented Wheat Germ Extract on Porcine Epithelial Cell Line Exposed to Deoxynivalenol and T-2 Mycotoxins

**DOI:** 10.1155/2020/3854247

**Published:** 2020-12-08

**Authors:** Judit Mercédesz Pomothy, Erzsébet Pászti-Gere, Réka Fanni Barna, Dorottya Prokoly, Ákos Jerzsele

**Affiliations:** ^1^Department of Pharmacology and Toxicology, University of Veterinary Medicine, 1078 Budapest, Hungary; ^2^Department of Physiology and Biochemistry, University of Veterinary Medicine, 1078 Budapest, Hungary

## Abstract

The effect of fermented wheat germ extract (FWGE) (*Immunovet®*) was evaluated with cotreatments with deoxynivalenol (DON) and T-2 toxin (T-2). These mycotoxins are produced by *Fusarium* mold species. The effects of FWGE on IPEC-J2 with DON and T-2 have not been studied until now. The IPEC-J2 porcine, nontumorigenic cell line was selected to investigate the outcome of the individually and simultaneously added compounds, as it has *in vivo*-like properties. The cells were treated for 24 h with the selected solutions; then, the IPEC-J2 cells were allowed to regenerate in a culture medium for an additional 24 h. In our results, DON and T-2 significantly increased the adverse impacts on cell viability and integrity of the cell monolayer. To elucidate the extent of oxidative stress, extracellular H_2_O_2_ concentrations and intracellular reactive oxygen species (ROS) were measured. FWGE appeared to be beneficial to IPEC-J2 cells given the separately and significantly decreased ROS levels. 1% and 2% FWGE could significantly reduce mycotoxin-induced oxidative stress. In conclusion, the results demonstrate that FWGE exerted protective effects to counteract the oxidative stress-provoking properties of applied fusariotoxins in the nontumorigenic IPEC-J2 cell line.

## 1. Introduction

The number of studies involving natural products and dietary supplements has shown rapid growth recently. Natural products contain extensive chemical diversity, which makes it difficult to replace the collection of naturally occurring molecules with synthetized drugs.

Wheat germ contains several bioactive ingredients, such as flavonoids, dietary fibres, as well as lignins, oligosaccharides, and vitamins [1]. Hidvegi et al. [2] demonstrated that wheat germ is rich in the glycosylated form of 2,6-dimethoxy-p-benzoquinone (DMBQ). The conversion of DMBQ into its biologically more active forms requires *β*-glucosidase enzyme [3]. Wheat germ is fermented by *Saccharomyces cerevisiae* yeasts [4] or treated with *Lactobacillus plantarum* dy-1 [5]. Fermented wheat germ extract (FWGE) is available in both human (*Avemar®*) and veterinary (*Immunovet®*) medicine. These products are aqueous extractions, which are fermented by *Saccharomyces cerevisiae*, and they contain several biologically active molecules [4, 6]. FWGE is applied as an adjuvant in human cancer therapy, because benzoquinones have antimetastatic [2], antimetabolic [6], antiangiogenic [7], and antiproliferative properties and are able to induce apoptosis [5, 8]. Furthermore, FWGE can enhance the cellular immune response [4, 9] and has an antioxidant effect [2].

It is of key importance worldwide to produce good quality feedstuff for livestock with the least amount of mycotoxin contaminants. *Fusarium* fungi are abundant in temperate climate zones and contaminate wheat and other cereals. This genus is capable of producing a wide variety of mycotoxins. One of these groups is the trichothecene mycotoxins, including deoxynivalenol (DON), nivalenol (NIV), T-2 (T-2) toxin, and HT-2 toxin [10, 11]. In general, these fusariotoxins generate reactive oxygen species (ROS) and interfere with the normal functions of mitochondria, which can lead to apoptosis. They also inhibit protein synthesis in eukaryotic cells [12], especially in epithelial and immune cells, where the rate of cell replication is high [13, 14]. Among farm animals, swine is the most sensitive species to fusariotoxin contamination; side effects are decreased feed intake, feed refusal, and vomiting [15].

The most frequently used cell line for oxidative stress-related studies are intestinal porcine epithelial cell line-1 (IPEC-1) and intestinal porcine epithelial cell line 2 (IPEC-J2), which are suitable for *in vitro* modelling of nontumorigenic epithelium. There are only few publications regarding IPEC-J2 cells treated with both DON and T-2 toxin. In these studies, transepithelial electrical resistance (TEER) decreased while cellular permeability was enhanced in parallel [16] by these toxic substances. Both DON and T-2 increase the ROS level intracellularly [17, 18].

The main objective of this study was to describe the beneficial effects of fermented wheat germ extract on the IPEC-J2 cell line induced with DON and T-2 mycotoxins. This study was focused on TEER and two oxidative stress markers: extracellular H_2_O_2_ and intracellular ROS productions.

## 2. Materials and Methods

### 2.1. Reagents

DON and T-2 were purchased from Merck (Darmstadt, Germany). Acetonitrile was obtained from Thermo Fisher Scientific (Waltham, MA, USA). The final concentration of acetonitrile in the cell culture medium was <0.5% (*v*/*v*). FWGE was diluted from a commercial product in powder form (Immunovet Pets, Immunovet Ltd., Hungary).

**Figure 1:**
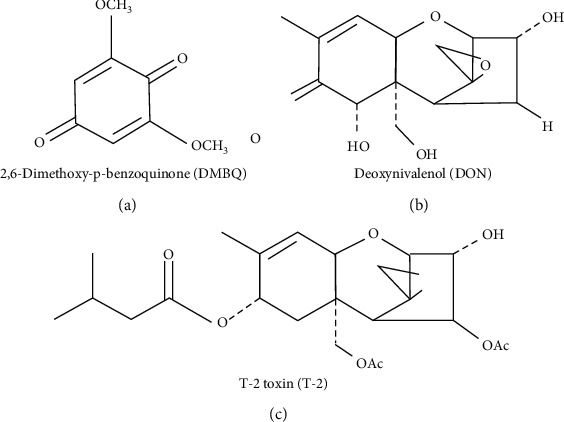
The chemical structures of (a) 2,6-dimethoxy-p-benzoquinone (DMBQ) from *Immunovet®* and the tested *Fusarium* mycotoxins, (b) deoxynivalenol (DON), and (c) T-2 toxin (T-2).

Prior to the experiments, cell viability studies were performed to select the working concentrations to DON, T-2, and FWGE (data not shown). 8 *μ*mol/L DON, 5 nmol/L T-2, and 1% and 2% FWGE concentrations were chosen from these results for further investigations (Figure 1).

### 2.2. Cell and Culturing Conditions

The porcine intestinal epithelial cell line IPEC-J2 (ACC 701) is nontumorigenic, intestinal columnar epithelial cells, which were isolated from neonatal piglet midjejunum. IPEC-J2 closely mimics *in vivo* pig and human physiology, which makes it a good model to study foodborne and plant-derived components.

This cell line form's polarized monolayers were maintained in 75 cm^2^ cell culture flasks with filtered caps (Orange Scientific, Braine-l'Alleud, Belgium) at 37°C in a humidified atmosphere of 5% CO_2_. The culture medium contains 50% Dulbecco's modified Eagle's medium (DMEM) and 50% Ham's F12 Nutrient Mixture (Merck, Darmstadt, Germany) supplemented with 1.5 mmol/L HEPES, 5% fetal bovine serum (Biocenter, Budapest, Hungary), 1% insulin/transferrin/sodium selenite medium supplement, 5 ng/mL epidermal growth factor, and 1% penicillin/streptomycin (all purchased from Invitrogen, Thermo Fisher Scientific, Waltham, MA, USA). Cells were used between passages 42 and 45. The media were changed every second day.

### 2.3. Experimental Design and Cell Treatments

To investigate the cell viability, the seeding density for the cells was 1 × 10^4^ cells/well of a 96-well plate (Transwell, Sigma-Aldrich, Corning Costar, Merck, Darmstadt, Germany). The cells were treated the next day after reaching a confluent state. When studying TEER, H_2_O_2_ production, and intracellular ROS levels, the cell-seeding density was 1.5 × 10^5^ cells/well in a 6-well polyester membrane insert (4.67 cm^2^) containing plates (Transwell, Sigma-Aldrich, Corning Costar, Merck, Darmstadt, Germany). These inserts were useful for the apically and basolaterally added treatments and for transepithelial electrical resistance measurements.

The stock solutions were freshly made with phenol red-free DMEM/F12 (Merck, Darmstadt, Germany). The DON and T-2 were diluted with acetonitrile (final concentration: <0.5% (*v*/*v*)); then, the following concentrations were made: 8 *μ*mol/L DON, 5 nmol/L T-2, and 1 and 2% final concentrations of FWGE. Cell cultures were exposed to the treatments for an incubation time of 24 hours; then, the IPEC-J2 cells were treated only with phenol red-free DMEM:F12 culture medium for an additional 24 h as regeneration. After the treatment and the regeneration, the TEER was measured, the cell-free supernatants were collected for extracellular H_2_O_2_ determination, and the DCFH-DA assay was added to the cells.

### 2.4. Evaluation of Cell Viability

Cytotoxicity was examined with an MTS reagent (CellTiter96 Aqueous One Solution, Promega, Bioscience, Budapest, Hungary) [19]. This test measures the rate of viable cells by determining the soluble tetrazolium salt conversion in the metabolically active cells to a coloured formazan product with the advantage over MTT that the solubilization step is not required for avoiding formazan precipitation in the aqueous medium.

IPEC-J2 cells were seeded in 96-well culture plates at 2 × 10^4^ cells/well and allowed 24 hours to reach confluence. Mycotoxin and FWGE solutions were added to the cells using a multichannel pipette and were incubated for 24 h at 37°C, 5% CO_2_. After the incubation time, the treatments were removed, and each well received 100 *μ*L of fresh phenol red-free medium containing 20 *μ*L of MTS solution. After an incubation time of 1 h at 37°C, the absorbance values were measured at 490 nm using an ELISA Plate Reader (EZ Read Biochrom 400, Cambridge, UK).

### 2.5. Determination of Cell Membrane Integrity

**Figure 2:**
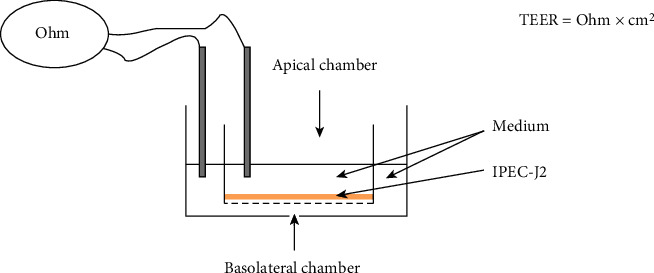
Schematic representation of TEER measuring method. Cell culture monolayers grown on polyester filter separate the apical and basolateral compartments.

The integrity of the IPEC-J2 cell monolayer can be followed by measuring transepithelial electrical resistance (TEER) between the apical and basolateral compartments of the IPEC-J2 cells (Figure 2). Cells were seeded to 6-well Transwell insert containing plates (polyester, 0.4 *μ*m pore size, Corning, Merck, Darmstadt, Germany), and the seeding density was 3 × 10^6^ cells/well. After the cells reached a confluent state, the barrier function was evaluated by measuring with an EVOM Epithelial Tissue Volt/Ohmmeter (World Precision Instruments, Berlin, Germany). 10 days after seeding, the IPEC-J2 monolayer achieved the 600 *Ω*/well values. The results were calculated as k*Ω* × cm^2^ by multiplying the values by the surface area of the monolayer (4.67 cm^2^). The high TEER value of IPEC-J2 monolayers grown on Transwell polyester filters demonstrates the functional integrity of the continuous cell association, acting as a single-layer tight physical barrier.

### 2.6. Detection of Changes in the Extracellular H_2_O_2_ Concentrations

Extracellular H_2_O_2_ production was monitored in IPEC-J2 cells by using the Amplex Red Hydrogen Peroxide Assay Kit (Invitrogen, Thermo Fisher Scientific, Waltham, MA, USA). The Amplex Red reagent reacts with H_2_O_2_ (in 1 : 1 stoichiometry) to produce a red fluorescent product called resorufin in the presence of horseradish peroxidase. After 24 and 48 h incubation time, 50 *μ*L of the cell-free supernatants was collected from the basolateral compartments and was mixed with the Amplex Red working solution according to the manufacturer's instructions. The fluorescence intensity was measured at 590 nm with a fluorometer using 530 nm excitation wavelength (Victor X2 2030, Perkin Elmer, Waltham, MA, USA).

### 2.7. Assessing the Changes in Intracellular ROS Levels

Measurement of disruptions in the intracellular redox state of IPEC-J2 cells was carried out using DCFH-DA dye (Sigma-Aldrich, Budapest, Hungary). DCFH-DA is oxidized to the highly fluorescent form of dichlorofluorescein (DCF) by the intracellular ROS [20]. Following a centrifugation process for 10 min at 10 000 rpm at 5°C, 100 *μ*L of cell-free supernatant was collected and pipetted into a 96-well plate. Samples of supernatant were collected at 24 and 48 h after treatments. The fluorescence intensities of the supernatants were measured at 530 nm with a fluorometer using 485 nm excitation wavelength (Victor X2 2030, Perkin Elmer, Waltham, MA, USA).

### 2.8. Statistical Analysis

The statistical analysis of the results was performed by using R Core Team (version of 2016) [21]. Differences between groups were analyzed by one-way ANOVA coupled with the post hoc Tukey test for multiple comparisons. ^∗^*p* < 0.05, ^∗∗^*p* < 0.01, and ^∗∗∗^*p* < 0.001 were considered to be statistically significant.

## 3. Results

### 3.1. Cell Viability Assessment Using MTS Assay

**Figure 3:**
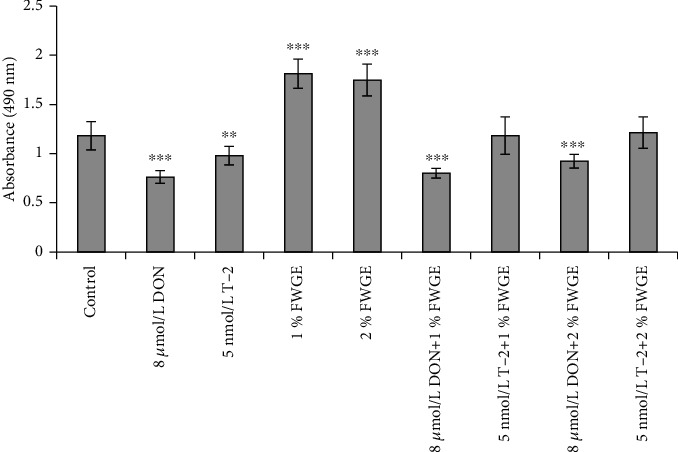
Cytotoxicity of 8 *μ*mol/L DON, 5 nmol/L T-2, and 1% and 2% FWGE and their combinations on IPEC-J2 cells at exposure of 24 h. ^∗∗^*p* < 0.01 and ^∗∗∗^*p* < 0.001 compared to the control values. Data are presented as means ± SD (*n* = 8).

IPEC-J2 cell viability was measured with the MTS reagent after a 24 h incubation time with 8 *μ*mol/L DON, 5 nmol/L T-2, and 1% and 2% FWGE. Their combinations were tested also: 8 *μ*mol/L DON+1% FWGE, 8 *μ*mol/L DON+2% FWGE, 5 nmol/L T-2+1% FWGE, and 5 nmol/L T-2+2% FWGE (Figure 3). According to absorbance values, 8 *μ*mol/L DON and 5 nmol/L T-2 significantly decreased cell viability (*p* < 0.001 and *p* = 0.0039), while 1% and 2% FWGE significantly increased the values of the treated cells compared to the control (both *p* < 0.001). The cytotoxic effect of 8 *μ*mol/L DON was not counteracted by simultaneous 1% and 2% FWGE treatments (*p* < 0.001). 1% FWGE added with 5 nmol/L T-2 did not change cell viability (*p* = 1.000) to the control level. The values of 5 nmol/L T-2+2% FWGE-treated cells showed no differences compared to control values (*p* = 0.999). Comparing the 5 nmol/L T-2 individual treatments with the concurrent 1% FWGE addition, significant differences in absorbances were observed (*p* = 0.0038). The 5 nmol/L T-2+2% FWGE showed significant differences compared to 5 nmol/L T-2 (*p* < 0.001).

### 3.2. TEER Measurements of the IPEC-J2 Cell Membrane Integrity

**Figure 4:**
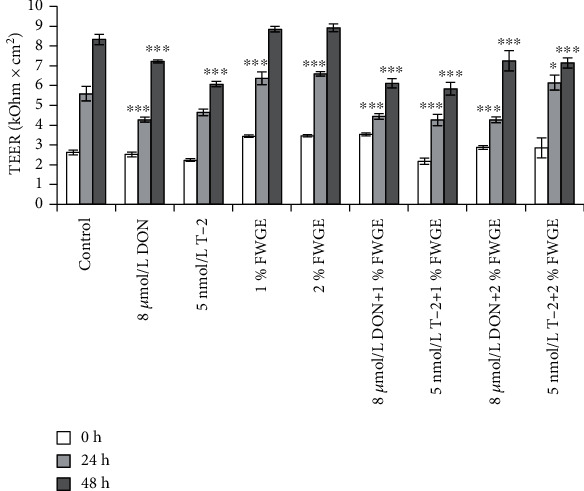
TEER measurements of IPEC-J2 monolayer. Prior to the experiments, TEER was measured (0 h). Cells were incubated with 8 *μ*mol/L DON, 5 nmol/L T-2, 1% and 2% FWGE, and the combination of these compounds for 24 and 48 h. ^∗^*p* < 0.05 and ^∗∗∗^*p* < 0.001 compared to the control values. Data are presented as means ± SD (*n* = 9).

To measure the changes in the integrity of the IPEC-J2 cell monolayer, TEER measurements were carried out prior to the treatments (0 h) after a 24 h treatment and after an additional 24 h (48 h) regenerative treatment (Figure 4). After treatments, 8 *μ*mol/L DON (24 h and 48 h) and 5 nmol/L T-2 (48 h) significantly decreased TEER values (both *p* < 0.001). In the case of the individually given FWGE (24 h), the TEER increased significantly (both *p* < 0.001). The 8 *μ*mol/L DON supplemented with 1% and 2% FWGE showed a significant decrease in TEER (both *p* < 0.001) compared to control values. 5 nmol/L T-2+1% FWGE (24 h) proved significantly reduced TEER values (*p* < 0.001); the 2% FWGE simultaneous treatment (24 h) indicated a significant increase (*p* = 0.018). After an additional 24 h regeneration treatment, the TEER values of 1% and 2% FWGE remained at the control levels (1% FWGE: *p* = 0.348; 2% FWGE: *p* = 0.194). In the case of 5 nmol/L T-2 added simultaneously with 1% FWGE and 2% FWGE, TEER decreased significantly (48 h, both *p* < 0.001).

### 3.3. Evaluation of H_2_O_2_ Concentrations from Cell-Free Supernatants with Amplex Red Assay

**Figure 5:**
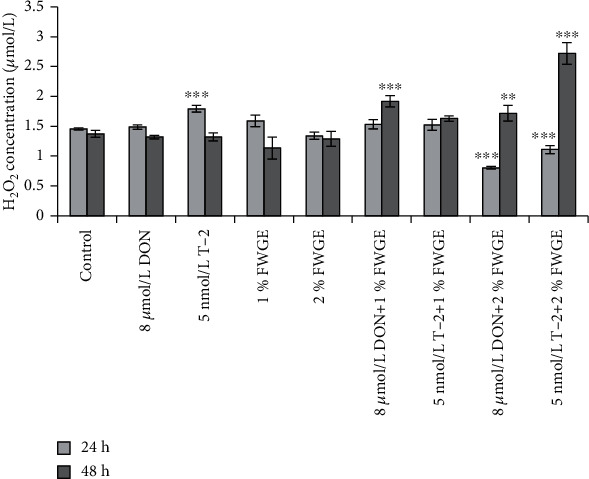
The changes of H_2_O_2_ concentrations after incubation of IPEC-J2 cells with 8 *μ*mol/L DON, 5 nmol/L T-2, 1% and 2% FWGE, and their combinations for indicated time periods (24 and 48 h). ^∗∗^*p* < 0.01 and ^∗∗∗^*p* < 0.001 compared to the control values. Data are presented as means ± SD (*n* = 8).

The changes in extracellular H_2_O_2_ concentrations were assessed after the 24 h treatment and an additional 24 h regeneration (48 h) in phenol red-free DMEM:F12 media (Figure 5). After a 24 h incubation with 8 *μ*mol/L DON, H_2_O_2_ concentrations remained unchanged (*p* = 0.070), while 5 nmol/L T-2 caused a significant increase (*p* < 0.001). The 1% and 2% FWGE treatments did not alter the H_2_O_2_ level (1% FWGE: *p* = 0.905; 2% FWGE: *p* = 0.705). When these compounds were added simultaneously, 8 *μ*mol/L DON+1% FWGE treatment did not cause a change compared to control treatment (*p* = 0.844), although 8 *μ*mol/L DON given with 2% FWGE significantly decreased H_2_O_2_ concentrations (*p* < 0.001). 5 nmol/L T-2+1% FWGE treatment did not differ significantly from the control treatment (*p* = 0.835), while the H_2_O_2_ level significantly decreased when 5 nmol/L T-2 was given at the same time with 2% FWGE (*p* < 0.001). After the regeneration period, the H_2_O_2_ concentrations of the mycotoxin-treated cells showed no differences to the control cells (DON: *p* = 1.00; T-2: *p* = 1.00). The H_2_O_2_ production of the priorly 1% and 2% FWGE-treated cells did not change after the regeneration (1% FWGE: *p* = 0.161; 2% FWGE: *p* = 0.996). After a 24 h regeneration period at the 8 *μ*mol/L DON+1% FWGE and 2% FWGE, the treated cells significantly increased the H_2_O_2_ concentration (*p* < 0.001 and *p* = 0.009). 5 nmol/L T-2+1% FWGE did not alter the H_2_O_2_ level compared to control-treated cells (*p* = 0.097). In contrast, 5 nmol/L T-2+2% FWGE significantly increased the H_2_O_2_ production after the regeneration period (*p* < 0.001).

### 3.4. Intracellular ROS Determination Using DCFH-DA Assay

**Figure 6:**
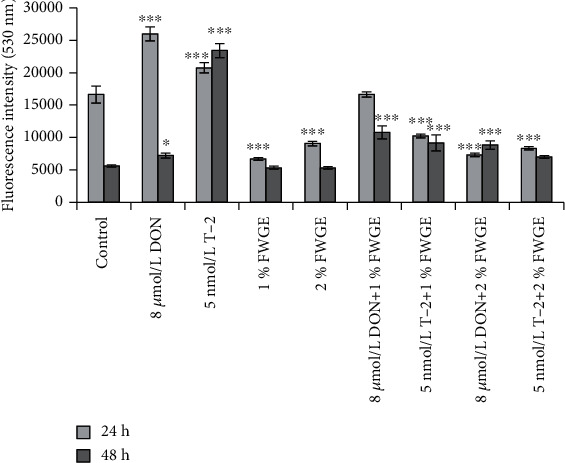
Intracellular ROS levels were measured after 24 and 48 h incubation with 8 *μ*mol/L DON, 5 nmol/L T-2, 1% and 2% FWGE, and their combinations. ^∗^*p* < 0.05 and ^∗∗∗^*p* < 0.001 compared to the control values. Data are presented as means ± SD (*n* = 6).

The DCFH-DA assay was used to estimate the intracellular ROS level present after 24 h treatments and additional 24 h regeneration (48 h) (Figure 6). After the treatment with 8 *μ*mol/L DON and 5 nmol/L T-2 for 24 h, the intracellular ROS were significantly higher compared to the control (both *p* < 0.001). The FWGE treatments significantly decrease the ROS levels intracellularly (both *p* < 0.001). 8 *μ*mol/L DON cotreated with 1% FWGE resulted in the same fluorescence intensities as the control-treated cells (*p* = 1.000), while 2% FWGE significantly reduced ROS production in cells exposed to DON compared to the control (*p* < 0.001). The cells given 5 nmol/L T-2 simultaneously with 1% FWGE and 2% FWGE showed significantly decreased values (*p* < 0.001). After the regeneration period, the prior 8 *μ*mol/L DON treatment significantly increased intracellular ROS (*p* = 0.043), similarly to the priorly 5 nmol/L T-2 treated cells (*p* < 0.001). The cells that were given 1% and 2% FWGE did not produce significantly different amounts of ROS intracellularly compared to the control (both *p* = 1.000). The ROS levels of the prior 8 *μ*mol/L DON+1% FWGE and 2% FWGE-treated cells were significantly higher (*p* < 0.001). The 1% FWGE cotreated with 5 nmol/L T-2 significantly increased fluorescence intensities (*p* < 0.001) after a 24 h regeneration, while 5 nmol/L T-2+2% FWGE-treated cells showed no differences compared to control cells (*p* = 0.142).

## 4. Discussion

According to Hernández et al. [22], the wheat germ extract's main active components include DMBQ, hydroxybenzoic acids, hydroxycinnamic acids, and apigenin. These naturally occurring compounds are glycosylated and physiologically not active [23]. There were only few experiments treating livestock animals with FWGE (*Immunovet®*). Three-week-old chickens were infected with *Mycoplasma gallisepticum* and treated with FWGE, and poultry remained clinically healthy [24]. In another research, FWGE was beneficial for the maintenance of general health conditions including biochemical and physiological parameters, increasing weight gain, and improved immune response to vaccination [25]. In growing pigs, FWGE enhanced weight gain and had a beneficial effect on cellular immunity. This effect is instrumental in promoting resistance against facultative pathogens [9]. As reported by Jerzsele et al. [26], 2% FWGE helped broiler chickens to gain greater body weight than the control group. It was also established that animals infected with *Salmonella Typhimurium* under controlled conditions and obtained FWGE treatments were not spreading the pathogens to other chickens.

A prominent issue in feed production is mycotoxin contamination. As major contaminants of cereals, DON and T-2 have been implicated in various gastrointestinal problems in farm animals, such as vomiting, feed refusal, diarrhoea [27], and oesophageal perforation as well as malabsorption [28]. DON can also be present in glycosylated form (for example, as DON-3-*β*-d-glycoside) in plants, which increases the toxicological effect of DON after consumption [29]. Before 2006, low-dose antibiotics were used to help the growth promotion of farm animals. These additional antibiotics were particularly not effective against mycotoxins but were beneficial to the general health status. Fortunately, numerous recent studies have been conducted in order to find a naturally occurring or plant-based solution to reduce the negative effects of mycotoxins and support the condition of farm animals. FWGE has several beneficial properties, such as its antioxidant effect [2], which can make this extract useful against oxidative stress generated by the two most common mycotoxins. Although mycotoxins negatively affect all farm animals, the swine is particularly sensitive to it.

The gastrointestinal epithelium is the first barrier for mycotoxin-contaminated feed. There are several *in vitro* studies regarding the impact of mycotoxins on epithelial cell metabolism, toxicity, and barrier integrity. Both DON and T-2 have demonstrated time- and concentration-dependent cytotoxicity. Szakács et al. [30] established that FWGE boosted the immune responses compared to T-2-treated weaned pigs. The IC_50_ for DON and T-2 was 23.52 *μ*mol/L and 20.4 nmol/L for 72 hours of incubation time on IPEC-J2 [16].

In this study, DON was added at 8 *μ*mol/L and T-2 at 5 nmol/L concentrations for 24 hours on IPEC-J2. Both DON and T-2 were shown to decrease the metabolic activity of the cells significantly. This result is in good correlation with that in the study by Sergent et al. [31]. The IC_50_ of DON was determined on the Caco-2 cell line at 2.22 *μ*mol/L, but a 0.67 *μ*mol/L concentration of DON inhibited the proliferation of cells for 48 hours [31]. In our studies, both 1% and 2% of FWGE increased cell viability compared to control cells. In the literature, FWGE was mostly examined on tumor cell lines where FWGE induced apoptosis and cell death in several cases [32]. 1 or 2% of FWGE did not preserve cell viability when 8 *μ*mol/L DON was added to the cells. In contrast, 1% and 2% FWGE enhanced the survival of IPEC-J2 cells treated simultaneously with T-2 compared to cells treated only with T-2.

IPEC-J2 cells can polarize and form a strong barrier through the development of tight junctions between cells [33]. The intercellular tight junction is the rate-limiting barrier in the paracellular pathway for permeation by ions and larger solutes. The TEER of cell monolayers can be considered a good indicator of the degree of organization of the tight junctions within the cell monolayer as well as that of epithelial integrity [34].

The authors did not find earlier results of studies using TEER to detect the effects of FWGE on the integrity of nontumorigenic intestinal cell monolayers exposed to fusariotoxins. On the other hand, TEER utilization in mycotoxin research has a more extensive representation in the literature. As reported by Goossens et al. [16] and Kang et al. [35], DON treatments significantly reduce the TEER values, depending on the dosage. Goossens et al. [16] also found that T-2 up to 210 nmol/L concentration for 72 h significantly lowered the integrity of the IPEC-J2 monolayer. Springler et al. [36] confirmed that DON reduced TEER significantly at 5–20 *μ*mol/L after 24 h incubation. Our study found that 8 *μ*mol/L DON and 5 nmol/L T-2 significantly reduced the TEER values during and after the treatments, while 1% and 2% FWGE alone significantly increased them. Based on our findings, 1% FWGE cotreatment with mycotoxins did not elevate the TEER, while 2% FWGE added for 24 h with 5 nmol/L T-2 helped the cells reach a higher TEER value.

Oxidative stress develops if concentrations of ROS exceed the antioxidant capacity of living entities. ROS are reactive species of radicals with a single unpaired electron, such as superoxide anion radical (O^2−^) and the hydroxyl radical (OH^−^), along with nonradical ROS such as hydrogen peroxide (H_2_O_2_). Sharply increasing intracellular ROS can cause oxidative stress with irreversible cell damage [37]. ROS can initiate the process of lipid peroxidation in the lipid membrane causing damage to the cell membrane's phospholipids and lipoproteins, and it can also damage DNA by propagating a chain reaction [38]. Moreover, oxidative stress mediated by ROS may increase cell apoptosis [39]. To counterbalance the prooxidant agents, the cells have intracellular nonenzymatic and enzymatic antioxidants, namely, tripeptide glutathione [40] and catalase, which can protect them from oxidative damage [41].

We examined oxidative stress by measuring extracellular H_2_O_2_ production and intracellular ROS generation in IPEC-J2 cells. We found that both 8 *μ*mol/L DON and 5 nmol/L T-2 significantly increased intracellular ROS levels during the 24 h treatment and after the 24 h regeneration. This is in agreement with the findings of Kang et al. [35] who published that DON at 6.7 *μ*mol/L in IPEC-J2 cells significantly elevated intracellular ROS levels after 24 h of mycotoxin exposure. Both 1% and 2% FWGE significantly decreased the ROS after a 24 h treatment. These findings are in good correlation with Karancsi et al. [42], who elucidated firstly beneficial effects of FWGE in case of LPS-evoked oxidative damage. FWGE could decrease excessive intracellular ROS levels after LPS administration and exerted protective effect on the integrity of the IPEC-J2 cell monolayer exposed to LPS treatment. Their study also showed that FWGE in different concentrations (1%, 2%, and 4%) did not affect cell death; moreover, FWGE in 2% concentration improved cell viability significantly after 24 h treatment. These results are seemingly contradictory to Otto et al. [6] who published that 24 *μ*mol/L DMBQ from FWGE inhibits cell cycle progress, induce apoptosis, and increase intracellular DCF fluorescence after a 24 h treatment in nine human cancer cell lines. Hidvegi et al. [2] resolved this contraversion by clarifying firstly that the effects of FWGE are not solely attributable to benzoquinones and secondly that IPEC-J2 is a nontumorigenic cell line.

When treated simultaneously, both 1% and 2% FWGE significantly decreased the DON- and T-2-mediated ROS levels. An interesting phenomenon was detected in terms of 2% FWGE as the extracellular H_2_O_2_ significantly decreased during the 24 h treatment; however, IPEC-J2 cells produced significantly higher H_2_O_2_ at the end of the regeneration period when cells were previously exposed to DON or T-2 toxins for 24 h.

## 5. Conclusions

In conclusion, 1% and 2% FWGE has favourable properties on IPEC-J2 cell lines as FWGE helps the cells to proliferate. 2% FWGE is a beneficial agent against intracellular ROS when treated with DON and T-2 simultaneously. To our knowledge, this is the first published report of FWGE cotreated with DON or T-2 in which TEER was utilized to determine the impact of FWGE on the integrity of the cell monolayer.

## Data Availability

The data used to support the findings of this study are available from the corresponding author upon request.
